# Cost-effectiveness of external cephalic version for term breech presentation

**DOI:** 10.1186/1471-2393-10-3

**Published:** 2010-01-21

**Authors:** Jonathan M Tan, Alex Macario, Brendan Carvalho, Maurice L Druzin, Yasser Y El-Sayed

**Affiliations:** 1 Department of Anesthesiology, Stony Brook University Medical Center, Stony Brook, New York, 11794-8480, USA; 2 Departments of Anesthesia, Stanford University Medical Center, Stanford, California, 94305, USA; 3 Health Research and Policy, Stanford University Medical Center, Stanford, California, 94305, USA; 4 Department of Obstetrics and Gynecology, Stanford University Medical Center, Stanford, California, 94305, USA

## Abstract

**Background:**

External cephalic version (ECV) is recommended by the American College of Obstetricians and Gynecologists to convert a breech fetus to vertex position and reduce the need for cesarean delivery. The goal of this study was to determine the incremental cost-effectiveness ratio, from society's perspective, of ECV compared to scheduled cesarean for term breech presentation.

**Methods:**

A computer-based decision model (TreeAge Pro 2008, Tree Age Software, Inc.) was developed for a hypothetical base case parturient presenting with a term singleton breech fetus with no contraindications for vaginal delivery. The model incorporated actual hospital costs (e.g., $8,023 for cesarean and $5,581 for vaginal delivery), utilities to quantify health-related quality of life, and probabilities based on analysis of published literature of successful ECV trial, spontaneous reversion, mode of delivery, and need for unanticipated emergency cesarean delivery. The primary endpoint was the incremental cost-effectiveness ratio in dollars per quality-adjusted year of life gained. A threshold of $50,000 per quality-adjusted life-years (QALY) was used to determine cost-effectiveness.

**Results:**

The incremental cost-effectiveness of ECV, assuming a baseline 58% success rate, equaled $7,900/QALY. If the estimated probability of successful ECV is less than 32%, then ECV costs more to society and has poorer QALYs for the patient. However, as the probability of successful ECV was between 32% and 63%, ECV cost more than cesarean delivery but with greater associated QALY such that the cost-effectiveness ratio was less than $50,000/QALY. If the probability of successful ECV was greater than 63%, the computer modeling indicated that a trial of ECV is less costly and with better QALYs than a scheduled cesarean. The cost-effectiveness of a trial of ECV is most sensitive to its probability of success, and not to the probabilities of a cesarean after ECV, spontaneous reversion to breech, successful second ECV trial, or adverse outcome from emergency cesarean.

**Conclusions:**

From society's perspective, ECV trial is cost-effective when compared to a scheduled cesarean for breech presentation provided the probability of successful ECV is > 32%. Improved algorithms are needed to more precisely estimate the likelihood that a patient will have a successful ECV.

## Background

Breech presentation at term occurs in 3-4% of pregnancies and is managed with elective cesarean, assisted vaginal delivery, or external cephalic version (ECV) [1,2]. Approximately 87% of breech presentations in the United States resulted in cesarean delivery [3], a significant increase from previous years [4,5]. Breech presentation is the third most common indication for cesarean and in some hospitals breech presentations are managed exclusively by cesarean delivery [6,7]. This may in part be due to the Term Breech Trial in 2000 that confirmed that breech presentations delivered by cesareans resulted in less neonatal mortality and short-term morbidity compared to vaginal breech delivery [8]. Maternal complications were also found to be similar for cesarean and vaginal delivery in the short [9] and long term [10,11].

Practice guidelines from the American College of Obstetrics and Gynecologists (ACOG) in 2006 recommend that decisions regarding the mode of delivery for breech presentation are dependent on the health care provider's experience. ACOG also suggests that cesarean deliveries are preferred over vaginal breech deliveries [12]. The desire to decrease the number of cesareans has renewed interest in ECV [13,14]. In fact, ACOG recommends ECV to lower the number of breech presentations [12,15]. ECV is indicated for women presenting with a confirmed singleton breech fetus at ≥ 36 completed weeks with no contraindications for vaginal delivery [16,17].

Quantifying the cost and effectiveness of ECV for breech presentation compared to scheduled cesarean is complicated because ECV does not always result in a cephalic presentation and subsequent vaginal delivery. While the probability of success in ECV is approximately 58% [18], the fetus may spontaneously revert back to breech before delivery [19]. In addition, vertex presentation at term does not necessarily translate to a vaginal delivery and a cesarean may still be performed for other reasons [20]. An unsuccessful ECV may warrant a second trial of ECV and potential adverse events with each trial, such as emergent caesarean section [21]. Patient preferences need to be factored into the decision.

Cost-effectiveness analysis produces a ratio. The numerator takes into consideration the additional costs that one intervention imposes over another. The denominator considers the incremental improvement in health-related quality of life calculated as quality-adjusted life-years (QALY). The goal of this study was to determine the incremental cost-effectiveness ratio, from society's perspective, of ECV compared to directly scheduling a cesarean delivery for term breech presentation. Our base case focuses on a parturient presenting with an ultrasound confirmed singleton breech fetus at ≥ 36 completed weeks gestation, with no contraindications for vaginal delivery. The base case represents a baseline scenario for the initial analysis before any of the assumptions are varied to elucidate key variables in the ECV decision.

## Methods

### Study Design

**Figure 1:**
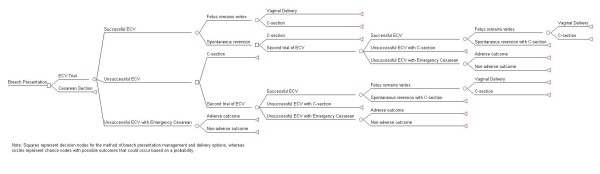
ECV Decision Tree Model*.

We developed a computer-based decision tree model using TreeAge Pro 2008 *(Tree Age Software, Inc., Williamstown, MA) *to evaluate the incremental cost-effectiveness of ECV (Figure 1). The primary outcome of the analysis was the incremental cost-effectiveness ratio, in 2007 U.S. Dollars per QALY. A societal perspective, was assumed as is commonly done to account for society having given up the opportunity to use those resources for some other purpose.

Also as commonly accepted, a threshold of $50,000 per QALY was used to determine cost-effectiveness. The time horizon captured in the study was 12-weeks after delivery which allowed for 6 weeks of recovery for vaginal delivery and 8 weeks for Cesarean. The 12-week time frame also allowed for maternal and neonatal adverse events and hospitalization times during delivery and recovery to be incorporated.

The main uncertainties are whether the ECV is successful in maneuvering the fetus to the vertex position, unsuccessful, or unsuccessful with the need for an emergency cesarean at the time of ECV. Following a successful ECV, further uncertainty exists between the fetus remaining vertex or spontaneously reverting back to breech. Following an unsuccessful, uncomplicated ECV, an elective cesarean delivery can be chosen or a second trial of ECV can be scheduled at another time. We modeled a different probability of success for the second ECV trial. Three modes of delivery were possible including, vaginal delivery, cesarean delivery or an emergency cesarean delivery. In addition, an emergency cesarean delivery may result in an adverse maternal or neonatal outcome necessitating ICU or NICU hospitalization

### Model Probabilities

We systematically reviewed the literature to determine the decision model probabilities for successful ECV on the first and second attempts, spontaneous reversion, mode of delivery and need for unanticipated emergency cesarean delivery (Table 1). We searched Medline, Cochrane and Up-to-Date for English-language peer-reviewed literature published between 1985 and 2008. Key words used in our search were: external cephalic version, breech, cesarean delivery, spontaneous reversion, and emergency cesarean section. We supplemented the search with a manual review of references from the retrieved articles.

**Table 1:** Estimated Probability (%) of Outcomes Utilized in Cost-Effectiveness Model

	Base Case Probability	(Range)	References
Successful ECV Procedure			
First Trial	58%	(35-86%)	[18]
Second Trial*	51%	(17-71%)	[22-24]
Consequence of ECV			
Fetus remains Vertex	-	-	
Cephalic Delivery with Successful ECV	-	-	
Cesarean After Successful ECV	27.60%	(8.5-30%)	[7,43,44]
Fetus Spontaneously Reverts to Breech *	6%	(3-10%)	[19,45-47]
Emergency Cesarean Needed at time of ECV	0.35%	(0-1%)	[21,48]
Adverse Outcome from Emergency Cesarean ^†^	1%	(0-1%)	

* Likelihood used in model base case is the weighted average of studies reviewed where the numerator and denominator across multiple studies were summed.

^†^Ranges of 0-1% were included in the sensitivity analysis even though no literature referenced these ranges in order to see the effect of both rare but expensive adverse events.

Where possible, probabilities were extracted from meta-analyses. If data were not available from a meta-analysis, probabilities were estimated by calculating the mean probability across the literature found in our review. This method allowed for studies to be weighed appropriately according to number of subjects. For example, the probability of successful ECV on the second trial used in our base case analysis was calculated to be 51%. This value was derived from three studies reporting repeat ECV success rates of 39.7% *(27/68) *[22], 71% *(77/108) *[23], and 17% *(6/36) *[24], where the numerators represent successful cases and denominators represent total number of subjects in their respective study. The ranges for our sensitivity analysis were derived from the upper and lower probability values identified in the literature review. In the example of the probability of successful second trial of ECV, the range used was 17-71%. Sensitivity analysis was useful in this case to determine the relative impact of changing probabilities on the outcome of our model, especially where the ranges reported in the literature were large.

### Determining Effectiveness

The primary measure of effectiveness was health-related quality of life calculated as QALY, which include a length of time component (e.g., 1 year) and a quality of life component (i.e., utility). The effectiveness measure was dependent on the assigned quality of life component (utility), the duration of the assigned quality of life and the total length of time captured in the model (12 weeks after delivery). Health utility is the numerical valuation of one's quality of life on a linear scale from 0.00 (death) to 1.00 (perfect health). For example, one QALY for an individual in perfect health (with a utility of 1.0) for 1 year is considered equivalent to 2 years in a health state with a utility of 0.5 (as might occur with a disability). Maternal health outcomes and fetal health outcomes were weighed equally.

Table 2 describes the utilities assigned to each of the maternal and neonatal health states. Four health states were used in the model including, perfect health, well health after vaginal delivery, well health after cesarean delivery and health during an adverse outcome due to an emergency cesarean. Maternal health states were independent of neonatal health states. Health states were estimated through observation and simulation of a mother and child experiencing each of the four health states used in the model.

**Table 2:** Utility Analysis Using Quality Adjusted Life Years (QALYs) for 12 Weeks

	Mean Utility	Expected Mean Duration*	QALY
**Maternal Health State**			
Perfect Health (Post-Recovery Phase)	1	-	1
Well Health after Vaginal Delivery	0.86	12 Weeks	0.97
Well Health after Cesarean Delivery	0.78	12 Weeks	0.95
Adverse Outcome Following Emergency Cearean (2-Day Hospitalization)	0.76	12 Weeks	0.93
**Neonatal Health State**			
Perfect Health (Post-Recovery Phase)	1	-	1
Well Health after Vaginal Delivery	1.00	12 weeks	1.00
Well Health after Cesarean Delivery	1.00	12 weeks	1.00
Adverse Outcome Following Emergency Cesarean (2-Day Hospitalization)	0.58	12 Weeks	0.90

*Duration of perfect health is dependent on 12 weeks minus the time spent in recovery

Maternal health quality of life was calculated with cesarean delivery as requiring a longer recovery time when compared to vaginal delivery. Direct measurement of utilities in patients recovering after delivery has not been performed. In such situations, the U.S. Panel on Cost-Effectiveness in Health and Medicine recommend the use of scale of valuations of states such as the EuroQol^© ^scale to be used [25]. For example, patients in the EuroQol^© ^EQ-5D scale are classified into one of five different health states (mobility, self-care, usual activity, pain, mood) [26]. Each state is scored from 1 (normal) to 3 (the most impaired). For example, a mobility score of "1" indicates "no problems in walking about," while a "3" is "confined to bed." The scores for the five states can be assigned a utility valuation from the general public. For example, a EuroQol^© ^mobility [3], self-care [3], usual activity [3], pain [2], mood [1] signifies a utility of 0.08. In contrast, EuroQol^© ^mobility [1], self-care [1], usual activity [2], pain [1], mood [2] signifies a utility of 0.65.

The expected quality of life for a vaginal delivery was based on the first 7 days at a utility of 0.50 and the remainder of the 6 weeks for recovery at a utility of 0.77. We assumed that the mother would subsequently return to perfect health at a utility of 1.00. Therefore, the mean utility following vaginal delivery was 0.86. The expected quality of life for a cesarean delivery was based on the first 21 days at a utility of 0.41 and the remainder of the 8 weeks for recovery at a utility of 0.77. We made the assumption that the mother would subsequently return to perfect health. Therefore, the mean utility following cesarean delivery was 0.78. The expected duration of health states during the recovery phase varied based on whether the mother and child were recovering from a vaginal or cesarean delivery. The recovery times for vaginal and cesarean delivery reflected the different standard time periods the law and society provides a mother to take off from work for recovery. Similar derivations to determine QALYs associated with each health state for the year following delivery were conducted for the neonate [27].

In the event of an adverse outcome following an emergency cesarean we assumed a 2-day hospital stay in the ICU or NICU was required at a mean utility of 0.76 and 0.75, respectively. The mean ICU or NICU utility for the mother and child was derived from an assumed 2-day ICU or NICU stay at a utility of 0.17 and 0.20 respectively, followed by utility of 0.60 for the subsequent 2 weeks post-delivery and a utility of 0.77 for the following 8 weeks until return to perfect health. We did not have to extrapolate QALY calculations beyond 12 weeks and therefore did not discount the utilities to adjust for future effects.

### Determining Costs

The cost of a medical service from society's point of view is the total net cost to all the different components of society. Direct hospital costs were included in the numerator of the cost-effectiveness ratio whereas indirect costs (or productivity costs) caused by morbidity or mortality were incorporated by the effectiveness measure, QALY, in the denominator. The use of costs and not charges as they appear on a patient's hospital bill is appropriate for our economic analyses because charges are known not to capture the actual economic consequences of an event. The use of costs from the perspective of society is further in accordance with recommendations by the U.S. Panel on Cost-Effectiveness in Health and Medicine [25].

Table 3 presents the costs of ECV, vaginal delivery, elective cesarean delivery and an emergency cesarean delivery. Each of the total costs were composed of the hospital cost, the professional cost of the obstetrician and anesthesiologists service, and the cost of the mother's time from a missed day of work according to the U.S. Census Bureau's 2003 median income for a full-time, year-round, female worker. We assumed the cost of a mother's missed day of work as an estimate of the opportunity cost of a mother participating in a procedure or hospital stay. Expected duration of procedure and length of stay were obtained from the US Department of Health and Human Services Healthcare Cost and Utilization Project data [28]. The calculated values are similar to previous studies [27,29,30].

**Table 3:** Estimated Costs (2007 U.S. Dollars) of ECV and Vaginal/Cesarean Delivery

	ECV	Vaginal Delivery	Elective	Emergency
			Cesarean Delivery	Cesarean Delivery
Mean hospital cost	$738	$3,605	$5,719	$5,719
Cost of obstetrician's professional service	$195	$1,587	$1,776	$1,792
Cost of anesthesiologist's service	-	$198	$228	$486
Cost of Mother's Time	$91	$191	$300	$400
				
Total	**$1,024**	**$5,581**	**$8,023**	**$8,397**

*Cost of Mother's time was itemized for each procedure expected duration/expected length of stay

Table 4 presents the derivation of the cost estimate for ECV. The cost estimates of ECV were determined by summing the cost of the individual components for the procedure. Physician professional costs were determined using the Current Procedural Terminology (CPT) codes reimbursed at the 2007 Medicare rate without geographic adjustment, such that, for example, the cost of the obstetrician's service for CPT 59412 (External Cephalic Version) equals $114. Similarly, calculations of cost of physician services were also obtained for CPT 59590 (routine obstetric care including antepartum care, cesarean delivery, and postpartum care). The cost of the anesthesiologist's service during a cesarean for example was also based on 2007 Medicare reimbursement rate of $20/unit. Using Medicare rates to estimate costs of physician services is the most common method used in health services research [27,28,30,31]. The cost of a labor nurse for the duration of the procedure anticipated to last 1.5 hour was estimated at $81.

**Table 4:** Itemized cost of ECV (2007 U.S. Dollars)

	Baseline Estimate ($)
Hospital costs	
Fetal heart rate monitoring ($53/hr × 1.5 hrs)	79.00
Electrocardiogram	65.00
Ultrasound scan ($86/scan × 2 scans)	172.00
Labor room ($116/hr × 1.5 hrs)	174.00
Blood Tests and drugs	175.00
Cost of Tocolysis (e.g., SQ terbutaline & abdomen lubricant)	25.00
Disposables	
Intravenous Kit Setup (e.g. Angiocath, Gauze, Tegaderm)	15.00
Saline solution	30.00
Swabs	3.00
Cost of obstetrician's professional service	
Physician	114.00
Labor Nurse	81.00
Cost of Mother's Time	91.00

Total Cost of ECV	1,024.00

The cost of an adverse outcome due to an emergency cesarean delivery was also included in the analysis to capture the cost of a rare but highly expensive event, from a financial and quality of life perspective. The cost of complications due to emergency cesarean delivery was estimated as maternal hospitalization of an additional $1,155 per additional ward day. The cost of complications due to emergency cesarean delivery for the neonate was assumed to cost $285 for a mild case of perinatal morbidity (2 hours of Neonatal intensive care unit monitoring + Perinatologist). Costs associated with maternal and neonatal morbidities assume successful treatment without subsequent complications. All costs were reported in 2007 United States Dollars. Future costs were discounted at 3% per annum in the base case scenario.

### Sensitivity Analyses

Sensitivity analyses were conducted to test a range (e.g., from low probability to the high probability of a particular event occurring) of values and determine their impact on the model. Ranges of probabilities tested can be found in Table 1. We conducted one-way and multi-way sensitivity analyses on both probabilities and costs across a range of values identified from the literature and cost calculations.

## Results

The estimated baseline cost for ECV equaled $1,024 (Table 4). Assuming a 58% chance of successful ECV, ECV resulted in an incremental cost-effectiveness ratio of $7,900/QALY in our base case when compared to scheduled cesarean (Table 5). This ratio is less than the commonly accepted threshold of $50,000 per QALY for an intervention. Therefore, ECV with a 58% likelihood of success can be considered cost-effective. Attempting a second ECV if the first failed was not cost-effective.

**Table 5:** Expected Costs per Strategy Using Baseline Values and Incremental Cost-Effectiveness Ratio

Delivery Strategy	Expected Costs ($)	Incremental Cost ($)	QALY	Incremental QALY	ICER* ($/QALY)
**Schedule Elective Cesarean**	**$8,023**	-	0.97	-	-
**Trial of External Cephalic Version**	**$8,102**	**$79**	0.98	0.01	**$7,900.00**

*ICER - Incremental Cost-Effectiveness Ratio

We found the decision model was only sensitive to the probability of successful ECV on the first trial. If the expected probability of successful ECV is less than 32%, then ECV costs more to society and has poorer QALYs for the patient. When the probability of successful ECV was between 32% and 63%, trial of ECV was cost-effective, meaning that the expected costs were higher than with scheduled cesarean delivery but with better outcomes. However, if the probability of successful ECV was greater than 63%, trial of ECV had less expected costs than cesarean delivery with better clinical outcomes.

The probability of conducting cesarean after successful ECV (range 8.5-30%), spontaneous reversion to breech (range 3-10%), probability of successful second trial of ECV (range 17-71%), emergency cesarean (range 0-1%) and adverse outcome from emergency cesarean (range 0-1%) did not affect the economics of the ECV decision so are considered not sensitive factors in the model. Furthermore, the model was not sensitive to variations in the cost of ECV (range $950-$1,200).

## Discussion

We computed the baseline cost for ECV to be $1,024, similar to that determined by Gifford et al [31]. From society's perspective, a trial of ECV at this general cost for our base case is cost-effective when compared to a scheduled cesarean delivery for breech presentation provided the probability of successful ECV is greater than 32%. Our hypothetical base case (a parturient presenting with a confirmed singleton breech fetus at ≥ 36 completed weeks gestation with no contraindications for vaginal delivery) is similar to the type of patient the ACOG recommends for trial of ECV [12,18]. When the probability of successful ECV is between 32% and 63%, trial of ECV costs more than cesarean but is still cost-effective because of the expected improved outcomes with ECV. This is possible because within this range, the incremental cost-effectiveness ratio for the ECV intervention was still less than the cost-effectiveness threshold of $50,000 per QALY. If the probability of successful ECV is greater than 63%, it is considered a dominant decision as a trial of ECV produces better outcomes and at less cost than a scheduled cesarean.

Although ACOG indicated the average probability of successful ECV to be 58%, the literature provides a range of reported success rates [18]. Since the cost-effectiveness of ECV hinges on the probability of successful ECV, results from this study are only as useful as the ability to determine the chance of a successful ECV being conducted on a specific patient. Maternal factors associated with an increased ECV success include higher parity [32,33], transverse-oblique presentations, posterior placenta [32], low uterine tone, non-frank breech presentations [33,34] and non-engagement of the presenting part [35]. Conversely, maternal factors associated with decreased ECV success rates include increased maternal weight [32] and advanced cervical dilation [36].

Other factors may also contribute to ECV success. A recent meta-analysis examining sixteen studies determined that epidural analgesia and tocolysis significantly improved ECV success rates when compared to controls [37]. However, the meta-analysis could not provide definitive evidence of a benefit from the use of spinal analgesia, vibroacoustic stimulation or transabdominal amnioinfusion [37]. Despite current knowledge of factors influencing probability of successful ECV [38], no single scoring system has been developed that accurately predicts ECV success [18].

Although several previous studies have assessed the economics of ECV, investigators did not incorporate quality of life as outcome measures in their findings [31,32,39]. Adams et al determined that if ECV was attempted, ECV saved about $3,000 per delivery [39]. Mauldin et al found that ECV saved $2,462 for each successful version [32]. Gifford et al assessed the delivery outcomes and cost of each method of delivery for breech presentation and determined that routine ECV practice can result in more vaginal deliveries and lower costs than strategies that do not include ECV [31]. These studies, however, were published before the results of the Term Breech Trial and therefore incorporated the use of assisted vaginal breech delivery in their management options as an alternative to ECV and cesarean delivery. Furthermore, the chance of an adverse outcome due to vaginal breech delivery or emergency cesarean was not incorporated into these different studies. Although the risk of adverse events, such as in the case of emergency cesarean is low, the high cost from a financial and quality of life standpoint should be incorporated into an economic analysis such as the one presented here. Vaginal breech delivery was not included in our model because of the demonstrated high risk of the procedure over cesarean breech delivery and it not being undertaken in common obstetric practice.

Limitations of this study are similar to those of any cost-effectiveness analysis. The cost-effectiveness for ECV as determined in this study is dependent on accurate estimates of probability and costs as factored into our computer model. To address this, we conducted sensitivity analyses for all probabilities and costs across the range of values found in our literature review. We consider the model robust since the probability of successful ECV, as we suspected a priori, was the key variable in the model.

Another limitation of this study was the use of a standard 6-week and 8-week time horizon for vaginal delivery or cesarean delivery respectively. This time horizon accounted for variations in recovery between vaginal delivery, cesarean delivery and an adverse delivery. However, this time horizon did not include longer-term health effects such as costs of future deliveries including repeated cesarean delivery and vaginal birth after cesarean. While these are important considerations, extending the time horizon further would significantly increase the complexity of the model as many other factors would need to be estimated.

Interestingly, the model was not sensitive to the probability of conducting a cesarean despite a successful ECV with no spontaneous reversion. This can be explained in part because the sum cost of ECV and subsequent vaginal delivery is similar to the cost of conducting a scheduled cesarean. The decision modeling also revealed that the cost of an adverse outcome associated with an emergency cesarean does not greatly impact the decision, which may be due to extremely low probability of an adverse outcome occurring offsets the potentially high cost of such an event [21].

This study examined the cost-effectiveness of ECV from society's perspective and not for a particular patient's preferences for delivery. Patient preferences should continue to play an important role in the delivery method for breech presentation. In fact, recent studies show the majority of women prefer a vaginal birth over a cesarean delivery, but would choose a cesarean if medically indicated [40-42]. Educating patients about potential risks in addition to potential benefits of a trial of ECV may help patients in their decision to balance both personal preference and medical needs.

## Conclusions

From society's perspective, ECV trial is cost-effective when compared to a scheduled cesarean for breech presentation provided the probability of successful ECV is > 32%. Improved algorithms incorporating maternal, clinician and procedure intervention factors should be developed to determine the probability a patient will have a successful ECV. Using a reliable and valid scoring system to predict the chance of ECV success can be combined with maternal preferences in clinical decision-making.

## Competing interests

The authors declare that they have no competing interests.

## Authors' contributions

All authors were involved in the study design and production of the manuscript. JT and AM were also involved in the analysis. All authors read and approved the final version of the manuscript.

## Pre-publication history

The pre-publication history for this paper can be accessed here:

http://www.biomedcentral.com/1471-2393/10/3/prepub
